# Role of Lipid Accumulation and Inflammation in Atherosclerosis: Focus on Molecular and Cellular Mechanisms

**DOI:** 10.3389/fcvm.2021.707529

**Published:** 2021-09-06

**Authors:** Khojasteh Malekmohammad, Evgeny E. Bezsonov, Mahmoud Rafieian-Kopaei

**Affiliations:** ^1^Department of Biology, College of Sciences, Shiraz University, Shiraz, Iran; ^2^Laboratory of Angiopathology, Institute of General Pathology and Pathophysiology, Moscow, Russia; ^3^Laboratory of Cellular and Molecular Pathology of Cardiovascular System, Institute of Human Morphology, Moscow, Russia; ^4^Institute for Atherosclerosis Research, Moscow, Russia; ^5^Department of Biology and General Genetics, I.M. Sechenov First Moscow State Medical University (Sechenov University), Moscow, Russia; ^6^Medical Plants Research Center, Basic Health Sciences Institute, Shahrekord University of Medical Sciences, Shahrekord, Iran

**Keywords:** atherosclerosis, LDL, cholesterol, inflammation, lncRNA, microRNA, medicinal plants

## Abstract

Atherosclerosis is a chronic lipid-driven and maladaptive inflammatory disease of arterial intima. It is characterized by the dysfunction of lipid homeostasis and signaling pathways that control the inflammation. This article reviews the role of inflammation and lipid accumulation, especially low-density lipoprotein (LDL), in the pathogenesis of atherosclerosis, with more emphasis on cellular mechanisms. Furthermore, this review will briefly highlight the role of medicinal plants, long non-coding RNA (lncRNA), and microRNAs in the pathophysiology, treatment, and prevention of atherosclerosis. Lipid homeostasis at various levels, including receptor-mediated uptake, synthesis, storage, metabolism, efflux, and its impairments are important for the development of atherosclerosis. The major source of cholesterol and lipid accumulation in the arterial wall is proatherogenic modified low-density lipoprotein (mLDL). Modified lipoproteins, such as oxidized low-density lipoprotein (ox-LDL) and LDL binding with proteoglycans of the extracellular matrix in the intima of blood vessels, cause aggregation of lipoprotein particles, endothelial damage, leukocyte recruitment, foam cell formation, and inflammation. Inflammation is the key contributor to atherosclerosis and participates in all phases of atherosclerosis. Also, several studies have shown that microRNAs and lncRNAs have appeared as key regulators of several physiological and pathophysiological processes in atherosclerosis, including regulation of HDL biogenesis, cholesterol efflux, lipid metabolism, regulating of smooth muscle proliferation, and controlling of inflammation. Thus, both lipid homeostasis and the inflammatory immune response are closely linked, and their cellular and molecular pathways interact with each other.

## Introduction

Atherosclerosis is an important cause of cardiovascular diseases such as ischemic heart disease and stroke. This complex multifactorial disease has chronic and progressive pathology characterized by lipid accumulation, low-grade inflammation in the walls of large- and medium-sized arteries, and endothelial dysfunction (1, 2).

Two key factors in the pathogenesis of atherosclerosis are cholesterol deposition and chronic inflammation (3). Atherosclerosis has three significant stages including fatty streak formation, induction of atheroma, and atherosclerotic plaques (4).

Reactive oxygen species (ROS) and reactive nitrogen species (RNS) damage the cellular function of lipids, proteins, and carbohydrates, and cause lipid peroxidation and low-density lipoprotein (LDL) oxidation (5). Oxidized LDL (ox-LDL) can remain in the vascular intima. ox-LDL has a crucial role in the initiation and development of atherosclerosis through inducing endothelial cell (EC) dysfunction, increasing leukocyte adhesiveness, inducing the expression of leukocyte and monocyte adhesion molecules on the endothelial surface such as Vascular Cell Adhesion Molecule-1 (VCAM), Intercellular Adhesion Molecule-1 (ICAM), E selectin and P-selectins (3, 6, 7). Monocytes, T lymphocytes, and mast cells are taken up into vascular wall intima by these adhesion molecules. T cells can respond to inflammatory signals by producing γ-interferon (IFN-γ) and lymphotoxin, and tumor necrosis factor β (TNF-β) (8, 9). Monocytes are converted into macrophages in the sub-endothelial space through Monocyte Chemotactic Protein-1 (MCP-1), Macrophage Colony-Stimulating Factor (M-CSF), and Interleukin-8 (IL-8). Macrophages uptake ox-LDL molecules via the scavenger receptor-A family to form lipid-laden foam cells. Yellow foam cells aggregate on the arterial walls and cause the development of fatty streaks (10, 11). A fibrous atherosclerotic plaque cap is formed from the fatty streak during the migration of Smooth Muscle Cells (SMCs) from media to intima and SMCs proliferation (9, 12). At advanced stages of atherosclerosis, Macrophages and T lymphocytes of fibrous atherosclerotic plaque cap secrete proteolytic enzymes such as metalloproteinase to reduce the stability of the fibrous cap and lyse the fibrous cap extracellular matrix. Breakdown of fibrous cap collagen content leads to coagulation process, blood clot formation, thrombus formation, and blockade of the arteries (3, 13, 14).

The pathophysiologic feature of atherosclerosis is an inflammatory, cellular, and metabolic process (4, 8). So, elucidating atherosclerosis pathogenesis is vital for understanding disease progression and the development of new therapeutics. This review will discuss the role and significance of inflammation and lipid accumulation especially LDL in the pathogenesis of atherosclerosis with more emphasis on cellular mechanisms. Furthermore, this review will highlight briefly the role of medicinal plants, long non-coding RNA, and microRNAs in the pathophysiology, treatment, and prevention of atherosclerosis.

## Sources of the information

The comprehensive information in this review article was obtained from noteworthy scientific databases, including Web of Science, PubMed, Science Direct, Scopus, and Google Scholar. The main search terms used in this study were atherosclerosis, LDL, lncRNA, microRNA, cholesterol, inflammation, and medicinal plants.

## Results

### Role of Lipid and Lipoprotein Accumulation in Atherosclerosis

Various forms of lipoproteins and lipids are implicated in lipid trafficking such as chylomicrons, sphingolipids, ceramides, very-low-density lipoproteins (VLDL), cholesterol, apolipoproteins, including ApoB (apolipoprotein B), intermediate-density lipoproteins (IDL), and low-density lipoprotein (LDL) (15–20). Lipid biomarkers are classic LDL and HDL, cholesterol, ox-LDL cholesterol, small dense LDL cholesterol, lipoprotein (a), and lipoprotein-associated phospholipase A2 (Lp-PLA2) (21).

Lipid homeostasis at various levels, including receptor-mediated uptake, synthesis, storage, metabolism, and efflux, as well as its impairment, are important for the development of atherosclerosis (22). Under normal physiological conditions, LDL in the cell can degrade in the lysosomes that prevent an imbalance of uptake, synthesis, efflux, and excessive lipid accumulation (23). The hypothesis of cholesterol retention in the arterial cells was suggested by Nikolai Anitschkow over 100 years ago (24). Enhanced plasma concentrations of cholesterol-rich apolipoprotein-B-containing lipoproteins are related to atherosclerosis. Lipoproteins can flux into and get out of the arterial wall via caveolin-1 and the scavenger receptor class B type I (SR-BI). Retention, or trapping, of cholesterol-rich apoB-containing lipoproteins within the arterial wall, is the key initiating event in atherogenesis. The retention of apoB-lipoproteins leads to lipid accumulation, triggers cellular responses within the artery wall, lesion development, maladaptive local responses, and plaque initiation (25, 26). The response-to-retention occurs via interacting lipoproteins with proteoglycans of the arterial wall (26). The consequences of the retention of apoB-lipoproteins include accumulation of lipids and exposure to local enzymes within the vessel wall (27–29). Important enzymes involved in apoB-lipoprotein retention, aggregation, and atherogenesis are secretory sphingomyelinase (S-SMase), lipoprotein lipase, and phospholipase A2. The most action of these enzymes is accelerating further retention of atherogenic lipoproteins (26). Also, retention of cholesterol-rich apoB-lipoproteins within the artery wall causes several modifications and significant biological consequences (26).

The major source of cholesterol and lipid accumulation in the arterial wall is proatherogenic mLDL (30). Desialylation, oxidation, formation of LDL self-associates, and LDL-containing immune complexes are the known LDL modifications (31). Also, all eight classes of scavenger receptors have been recognized as both native and modified LDL (32).

The first known atherogenic LDL modification is desialylation, and the trans-sialidase (neuraminidase) enzyme is responsible for LDL desialylation. The action mechanisms of intracellular lipid accumulation by desialylated LDL are performed in two ways; the first is binding, uptake, and degradation of LDL. The second is the evaluation of hydrolysis and esterification rates of lipids in LDL particles (33). LDL particles desialylation causes autoantibodies production, which forms circulating immune complexes (CIC) containing LDL (34). CIC causes the secretion of pro-inflammatory cytokines and macrophages apoptosis (34). Desialylated LDL causes the following consequences: lipid accumulation, enhancing its binding to the arterial proteoglycans, proliferative activity, connective tissue matrix components synthesis, breaking intercellular communication, chronic inflammation, and intracellular esterification of free cholesterol through preventing of the cholesterol acyltransferase esterifying activity in macrophages and cholesterol accumulation (24, 34).

Oxidative lipoprotein modification by intimal oxidizing agents, proteases, and lipases leads to the generation of oxidized phospholipids (oxPLs), inducing leukocyte recruitment, leukocyte activation, LDL aggregation, formation of cholesterol crystals, and inflammation (16).

Modified lipoproteins, such as ox-LDL, and LDL binding with proteoglycans of the extracellular matrix in the intima of blood vessels, cause aggregation of lipoprotein particles, endothelial damage, leukocyte recruitment, and inflammation (26). Then, foam cell forms from aggregated modified apoB-lipoproteins through taking up oxidized, proteolyzed, or lipolysed lipoproteins, taking up cholesterol crystals by macrophages and taking up lipoproteins by SMCs via different classes of scavenger receptors (SR), such as SR class A (SR-A1, also known as CD204), SR class B (CD36), and the lectin-like oxidized LDL 1 receptor (LOX-1, or SR-E1), which can identify and bind to modified lipoproteins (35–37). Also, aggregated mLDL may accumulate in macrophages, which is recognized by low-density lipoprotein receptor-related protein 1 (LRP1) and Toll-like receptor 4 (TLR4) or degraded via lysosomal synapses (38–40).

SR-A is expressed on the surface of macrophages by regulating various factors. Sac1 and Sac3 phosphatases maintain a constant level of SR-A expression in the endoplasmic reticulum (41). Upregulation of Sac1 expression increases the SR-A receptor abundance. Tumor Necrosis Factor-alpha (TNF-α) and Interleukin-6 (IL-6) can upregulate the SR-A expression leading to LDL accumulation by macrophages (42).

CD36 with its ligands (mLDL, HDL, fatty acids, and VLDL) is involved in lipoprotein uptake and lipid metabolism (43). Signaling pathways, such as non-receptor tyrosine kinase (SRC), cJun NH2-terminal kinase (JNK), Rac (GTPase) protein, and nuclear factor-kB (NF-kB transcription factor), are activated by interacting mLDL and CD36, which can result in LDL absorption, oxidative processes, and the production of proinflammatory cytokines (32, 44). LOX-1 from the E class SR family is involved in lipid accumulation, and its expression is increased in the intima under the inflammation and oxidative condition (32).

In the context of the response-to-retention, HDL has the following roles: interfering with the irreversible binding of plasma LDL to arterial wall proteoglycans, blocking SMase-induced aggregation of LDL, omitting toxic lipids, ameliorating the maladaptive inflammatory infiltrate, inhibiting lipoprotein oxidation, EC protection and suppression of monocyte adhesion (45–49).

### Role of Inflammation in Atherosclerosis

The role of inflammation in the pathogenesis of atherosclerosis was suggested in 1908 by Sir William Osler (50). Inflammation participates in all phases of atherosclerosis. For example, stable plaques and ruptured plaques are characterized by a chronic inflammatory infiltrate and “active” inflammation in the thinning of fibrous caps (21). Inflammation is linked with different risk factors of atherosclerosis (26, 51). All risk factors of atherosclerosis cause inflammatory response (9). Cellular cholesterol and inflammation can affect the immune system and autoimmune diseases (26).

Ox-LDL and generally modified lipoproteins increase endothelial damage, leukocyte recruitment, and inflammation. Several studies revealed that high levels of E-selectin, ICAM-1, and VCAM-1 are expressed by inflamed endothelium (52). Thus, these activated endothelial cells are the local source of leukocytes recruited into an atherosclerotic lesion (21). One of the earliest signs of atherosclerosis is endothelial dysfunction or activation (53). High inflammatory responses lead to arterial wall thickening (53).

Various inflammatory cells such as macrophage foam cells and T lymphocytes participate in inflammatory responses and the progression of atherosclerosis (21). Different cytokines are produced by macrophage foam cells leading to activation of SMCs and extracellular matrix production (8).

In the initiation phase of atherosclerosis, monocytes and lymphocytes migrate into the inner arterial wall with the help of MCP-1 and T cell chemo-attractant. Inside the intima, monocytes are differentiated into macrophage foam cells under the influence of M-CSF. All activated cells release inflammatory cytokines and proinflammatory mediators. In the fatty-streak lesion, T-cells secrete TNF-β, IFN-γ, fibrogenic mediators, and growth factors that can cause the migration and proliferation of smooth muscle cells. Also, activated T-cells cause the following important inflammatory reactions: stimulating Matrix Metalloproteinase (MMP) production by macrophages in the lesion, producing IFN-γ, and halting collagen synthesis by the SMCs. It is considered that plaque formation is related to increased plasma concentration of C-Reactive Protein (CRP) (53). As mentioned above, inflammatory cells, macrophage foam cells and T-cells, and proinflammatory mediators (cytokines, interleukins) have important roles in all different stages of atherosclerosis.

Traditional risk factors of atherosclerosis are Hypercholesterimia, HDL, hypertension, obesity, and diabetes. The high level of LDL cholesterol in the blood causes artery and vascular smooth muscle cells (VSMCs) injury, induction of adhesion molecules and proinflammatory cytokines expression in macrophages and endothelial cells, and activation inflammatory response by expressing mononuclear leukocyte recruiting mechanisms (9).

The mechanisms of actions of HDL are inhibiting LDL oxidative modification, blocking the proinflammatory effects of ox-LDL, promoting antioxidant enzyme activity such as acetylhydrolase and paraoxonase, which neutralize oxidized lipids, and proinflammatory effects (9).

Angiotensin II (AII), as a powerful vasoconstrictor, is produced during hypertension (54). AII increases the growth of SMCs and facilitates smooth muscle lipoxygenase activity and results in speeding up inflammation. Also, it causes inflammation of endothelial intima via promoting the expression of cytokines (IL-6) and MCP-1 and superoxide anion production by the endothelium and SMCs of the artery (9, 54). In fact, Ang-II has pro-atherogenic effects and increases endothelial oxidative stress. Also, it up-regulates the LOX-1 gene and causes the activation of apoptosis pathways and induction of endothelial dysfunction (54).

Oxidative stress, ROS formation, endothelial activation, and disruption of cellular defense systems in conditions of chronic hyperglycemia and diabetes promote inflammation. Glycated lipoproteins protect the proinflammatory action of cytokines in the arterial endothelium (55, 56).

The elevated levels of VLDL and inflammatory processes can initiate atherosclerosis. Adiponectin, leptin resistin, TNF-α, and IL-6 are cytokines generated from adipose tissue, which can impact inflammation (9). Biomarkers of inflammation or indicators of the inflammatory response are CRP, Protease Activated Receptor (PAR), CD40, Interleukin-18, LOX-1, and Lipoprotein-associated phospholipase (Lp-PLA2) (57). Obesity accelerates atherosclerosis through increasing glucose level, abnormal lipid profile, and systemic inflammation (58).

One of the most stable plasma biomarkers for low-grade systemic inflammation is CRP. It is a valuable tool for predicting, diagnosing, and prognosis of atherosclerosis. CRP has a direct role in promoting the inflammatory component of atherosclerosis. The significant mechanisms actions of CRP are: downregulating endothelial nitric oxide synthase (eNOS) to prevent nitric oxide releasing into the endothelium, destabilizing eNOS mRNA, increasing the release of endothelin (ET-1), adhesion molecules (VCAM-1, ICAM), MCP-1, migration of SMCs and facilitating LDL uptake by macrophage (59).

PAR has four types (PAR-1,−2,−3,−4). PAR-1 activation facilitates the binding of monocytes and leukocyte recruitment in the endothelium. PAR-1 and PAR-2 can enhance the leukocytes and platelets to the endothelium. It is proposed that the proinflammatory property of PARs is induced by IL-1 and TNF-α in inflamed cells (9).

CD40/CD40L is a proinflammatory system belonging to the TNF family. This protein is found in atherosclerotic plaques and expressed by activated macrophages, SMCs, vascular endothelial cells, and T lymphocytes. Binding of soluble CD40L (sCD40L), derived from activated platelet, to CD40 on SMC, and endothelium causes endothelial dysfunction, inflammation, and production of proinflammatory cytokines (IL-6, IL-1), VCAM-1, ICAM, MCP-1, MMPs, fibroblast growth factor, vascular endothelial growth factor, platelet activation, and thus the production of ROS and RNS (9, 60–62).

Interleukin-18 (IL-18) is made by monocytes and macrophages and its receptor is expressed on T lymphocytes (T helper). IL-18 binds to its receptors and causes inflammation and plaque formation via producing IL-1, TNF-α, and a positive feedback mechanism. The stability of plaque is reduced by increasing MMP expression (9). This proinflammatory cytokine causes induction of IFN-γ, inhibiting collagen synthesis, preventing thick fibrous cap formation, and facilitating plaque destabilization (63).

LOX-1 is found in endothelial cells, macrophages, and SMCs. It is bound only to ox-LDL and detected in all phases of atherosclerosis. The ox-LDL/LOX-1 complex causes the following impacts: increasing ROS production, death of SMCs, MMP influence on a fibrous cap, providing a route of entry for ox-LDL into endothelium, disrupting the normal endothelial function and monocyte adhesion, and infiltration. The stimulation of LOX-1 causes endothelial dysfunction, leukocyte adhesion, collagen degradation, and foam cell formation (64). Lp-PLA2 has performed its proinflammatory and proatherogenic functions by promoting monocyte chemotaxis, enhancing expression of mononuclear leukocyte adhesion molecules in endothelial cells, producing lysophosphatidylcholine (lysoPC) and non-esterified fatty acid moieties (9).

### The Cellular Mechanisms of Inflammation and Lipid Accumulation in the Pathogenesis of Atherosclerosis

The intimal layer of the arterial wall is the location of the Atherosclerotic lesion. The endothelial cells separate the intima from the lumen of the vessel. Under the basal membrane, different types of cells such as macrophages, dendritic cells, foam cells, lymphocytes, and other inflammatory cells are found in intimal atherosclerotic lesions (65). Deeper layers include SMCs and pericytes that participate in immunity reactions. Pericytes secrete pro-inflammatory cytokines such as IL-1, IL-6, and TNF. They also act as phagocytes and antigen-presenting cells (66, 67). Lesion development is associated with a local enhancement of the number of macrophages, loss of intercellular communication, and changes of Pericytes, macrophages, and SMCs phenotype (68).

Lipid droplets accumulate in the cytoplasm of Pericytes, macrophages, and SMCs and lead to change their appearance. The presence of Pericytes, macrophages, and SMCs in the subendothelial space of the arterial wall is an early manifestation of atherosclerosis (68). Atherogenic mLDL is the primary source of lipids that are found in foam cells and the circulation of atherosclerotic patients. Atherogenic modification of LDL is mentioned in section 3.1. mLDL stimulates phagocytosis by pericytes and macrophages. Then inflammatory cytokines are secreted, which causes the attraction of immune cells to the location of the inflammation. Inflammatory cytokines cause the accumulation of intracellular lipids. Intracellular lipids accumulation leads to rupture of the cells (68). Also, increasing proliferative activity and stimulation of extracellular matrix synthesis are occurring in the phase of inflammatory reaction (69). Based on current consensus, endothelial activation and enhanced permeability is the key event in the atherosclerotic lesion development. Endothelial cells express cytokines and chemokines (IL-1, TNF-α, MCP-1, growth factors, and adhesion molecules). This leads to the interaction of circulating immune cells with endothelium and enhancement of the pro-inflammatory signaling at the emerging lesion site (68). As a result of the increase of pro-inflammatory cytokines, endoplasmic reticulum stress in the arterial wall cells and apoptosis have occurred (70). This process and cytokine-induced inflammation lead to interrupt the normal activity of mitochondria and then impaired mitophagy and apoptosis (71). mtDNA mutations have an important role in atherosclerosis. These mutations lead to impaired glucose and fat metabolism, increased oxidative stress, ROS generation, and cell death (68). ROS act as modulators of gene expression related to atherosclerosis development. The mutation spectrum of the mitochondrial genome is useful for the early detection of atherosclerosis (3, 71–74).

Lysosome function is linked to inflammatory cytokine release and regulation of immune response. So, loss of lysosomal function in ox-LDL-loaded Macrophages is a general effect related to the excess lipid loading during atherosclerosis (75, 76).

### The Role of Long Non-coding RNAs in Lipid Accumulation and Inflammation

Long non-coding RNAs (lncRNAs) have been considered as a novel group of epigenetic regulators with significant roles in the pathogenesis and development of atherosclerosis. Also, these biomarkers have the potential for targeting them therapeutically (77). lncRNAs have multiple functions in a wide range of biological processes. They are involved in regulating macrophage, lipid metabolism and inflammatory, and immune responses (78, 79).

Taurin-up-regulated gene 1 (TUG1) knockdown prevents hyperlipidemia and atherosclerotic lesions through up-regulating the miR-133a expression which targets the fibroblast growth factor 1 (FGF1) (80, 81). LncRNA-H19 has influenced lipid metabolism by targeting miR-130b. It suppresses lipid metabolism and increases lipid accumulation which causes lipid metabolic disorders and atherosclerosis (79, 82). lncRNA-H19 knockdown decreases inflammatory responses and pro-inflammatory factors (IL-1β, IL-6, and TNF-α) and enhances the expression of anti-inflammatory factors (IL-4 and IL-10). So, H19 can prevent endothelial inflammation by inhibiting the STAT3 (signal transducer and activator of transcription 3) signaling pathway (82–84). lncRNA RP5-833A20.1 has a regulatory function in lipid metabolism and inflammation during atherosclerosis. Its target is miR-382. This lncRNA causes enhancement of inflammatory cytokines (TNF-α, IL-1β, and IL-6) and decreases cholesterol efflux via the miR-382-mediated Nuclear factor I A (NFIA) pathway, and attenuates ATP binding cassette transporter A1 (ABCA1) levels (85). lncRNA E330013P06 is a new pro-inflammatory long non-coding RNA. MiR143/145 is a key target of this lncRNA. Overexpressing E330013P06 promotes foam cell formation via increasing inflammatory genes (Nos2, Il6, and Ptgs2) and scavenger receptor CD36 (78). lncRNA growth arrest-specific 5 (GAS5) regulates atherosclerosis developments via various mechanisms including promoting monocyte migration, interaction with the histone methyltransferase EZH2 (enhancer of zeste homolog 2) PRC-2 subunit, decreasing ABCA-1 expression, cholesterol efflux, and producing of inflammatory cytokines via targeting miR-221 and up-regulating MMP-2 and MMP-9 (86, 87). Antisense non-coding RNA in the INK4 locus (Anril), or CDKN2B antisense RNA 1 (CDKN2B-AS1), has different effects on lipid metabolism and inflammation in atherosclerosis. It can promote lipid uptake and LPS induced-inflammation via regulating the CDKN2B promoter and activating the NF-kB signaling pathway (4, 88, 89). Knocking down of long non-coding RNA Maternally Expressed Gene 3 (MEG3) protects the VSMCs from ox-LDL-induced injury by enhancing p53 expression (90). Also, it increases pyroptosis by sponging miR-223 and targeting NOD-like receptor protein 3 (NLRP3). Also, the ox-LDL-induced apoptosis in VSMCs by sponging the miR-361-5p. So, MEG3 promotes the development of atherosclerosis by increasing inflammation (91–93). Long non-coding RNA-DAPK1-IT1 decreases ABCA1 and ATP-binding cassette transporters G1 (ABCG1) protein levels in THP-1 macrophages by sponging miR- 590–3p and targeting lipoprotein lipase (LPL). Thus, it reduces the levels of HDL and enhances the levels of LDL (94).

**Figure 1:**
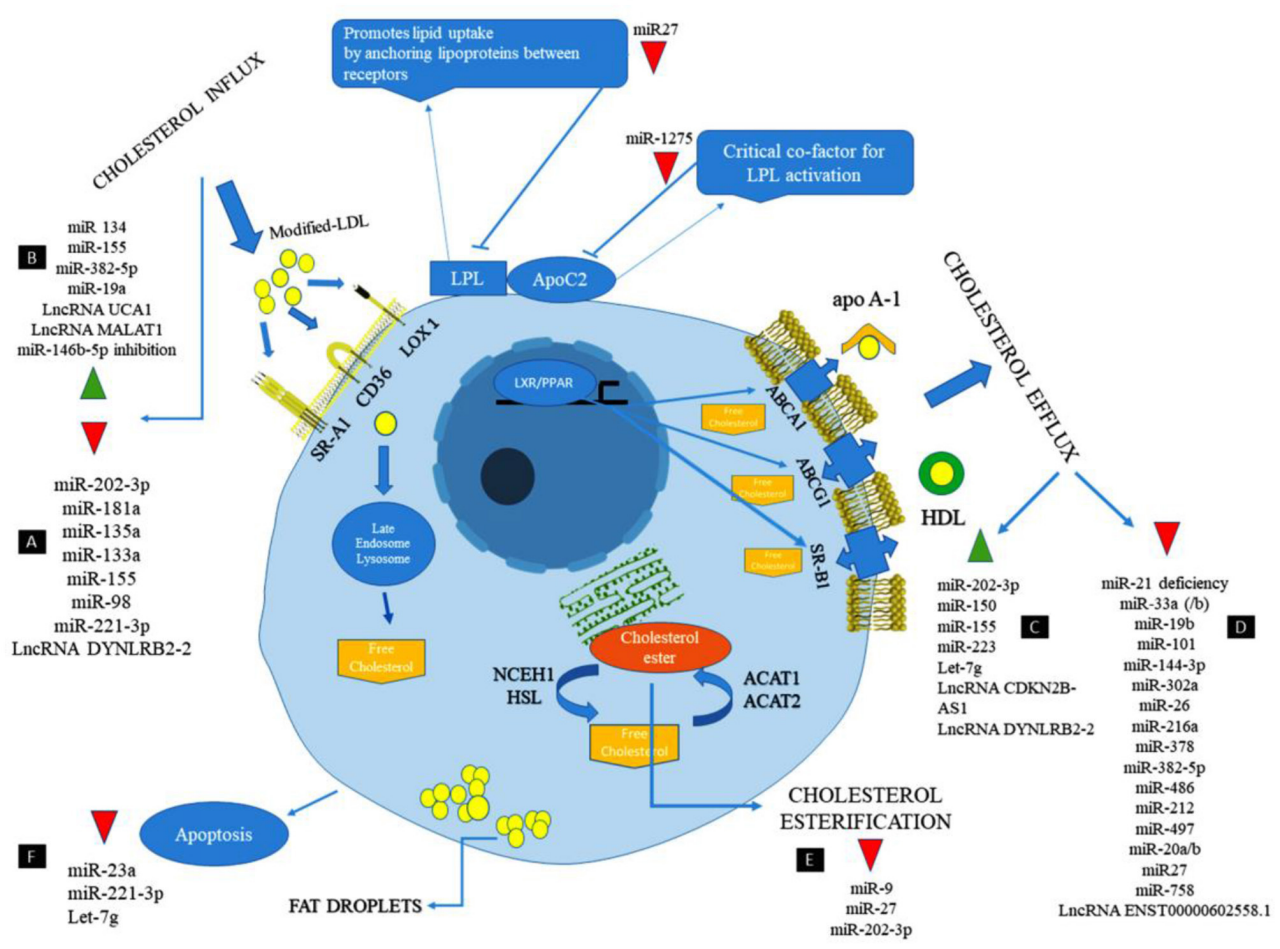
The cellular mechanism of atherosclerosis and the involvement of non-coding RNAs in this process (107).

Long non-coding RNA metastasis-associated lung adenocarcinoma transcript 1 (MALAT1) has a protective effect against atherosclerosis lesions. The supportive effects of MALAT1 against the ox-LDL-induced apoptosis were performed through these action mechanisms: Upregulating endothelial-to-mesenchymal transition (EndMT), competing with miR-22-3p, induction of autophagy by inhibiting the PI3K/AKT pathway or sponging miR-216a-5p, and suppressing the production of ox-LDL mediated pro-inflammatory cytokines such as IL-6 and IL-8 via sponging miR-155 (95–98). The anti-inflammatory effect of MALAT1 is increasing lipid uptake in macrophages via interacting with nuclear enriched abundant transcript (NEAT1) (99, 100). lncRNA myocardial infarction associated transcript (MIAT) has a protective effect against the ox-LDL-induced apoptosis through inhibiting miR-181b and signal transducer and activator of transcription 3 (STAT3) (101, 102). From a therapeutic point of view, lncRNA-DYNLRB2-2 has anti-atherosclerotic properties via different mechanisms, including promoting cholesterol efflux, inhibiting inflammation, increasing ABCA1 expression, inhibiting THP-1 macrophage foam cell formation, activating the LKB1/AMPK/mTOR signaling pathway, and inhibiting the lipopolysaccharide (LPS)-induced inflammatory cytokines such as TNF-a, IL-1b, and IL-6 in macrophages by reducing TLR2 expression (4, 103–105). From a therapeutic point of view, Anril causes cholesterol efflux and reduction of inflammatory cytokines such as IL-1b and TNF-α in ox-LDL-exposed THP-1 macrophages via inhibiting a disintegrin and metalloprotease (ADAM) expression (106). The most important lncRNAs involved in lipid accumulation and inflammation are summarized in Table 1 and Figure 1.

**Table 1:** A list of lncRNAs involved in lipid accumulation and inflammation.

**lncRNA**	**Biological processes**	**Target**	**Function**	**Reference**
MALAT1	Leukocyte activation, cholesterol metabolism	miR-216a-5p, miR-155 and miR-22-3p	Inhibiting inflammation	(95–100)
MIAT	Macrophage apoptosis	miR-181b/STAT3	Apoptosis, inhibiting inflammation	(101, 102)
GAS5	Cholesterol metabolism	miR-221, MMP-2 and MMP-9	Promoting inflammation	(86, 87)
DYNLRB2-2	Cholesterol metabolism	ABCA1 and TLR2	Inhibiting inflammation and increasing cholesterol efflux	(4, 103–105)
Anril	Endothelial dysfunction; cholesterol metabolism	ADAM10 and CDKN2B	Promoting lipid accumulation and inflammation	(4, 88)
DAPK1-IT1	Cholesterol metabolism	miR- 590–3p and LPL	Promoting lipid accumulation and inflammation	(94)
MEG3	Lipid metabolism	miR-223 and miR-361-5p	Increasing inflammation and pyroptosis	(90–93)
E330013P06	Lipid metabolism	miR-143 and miR-145	Promoting inflammation and foam cell formation	(78)
RP5-833A20	Cholesterol metabolism	miR-382	Promoting inflammation/decreasing cholesterol efflux	(85)
H19	Endothelial dysfunction	miR-130b	Increasing lipid accumulation, and Inflammation	(82–84)
TUG1	Endothelial dysfunction; Macrophage apoptosis	miR-133a/FGF1	Promoting lipid accumulation, inflammation and apoptosis	(80, 81)

### Role of microRNAs in Lipid Accumulation and Inflammation

MicroRNAs have appeared as key regulators of several physiological and pathophysiological processes in atherosclerosis including regulation of HDL biogenesis, cholesterol efflux, lipid metabolism, regulating of smooth muscle proliferation, and controlling of inflammation through the activation and infiltration of leukocytes via the vascular wall (108–110). Moreover, numerous studies have demonstrated the beneficial role of miRNAs as therapeutic targets in the treatment of atherosclerosis and cardiovascular disease (Table 2 and Figure 1) (131).

**Table 2:** A list of microRNAs involved in lipid accumulation and inflammation.

**miRNA**	**Target**	**Function**	**Reference**
miR-155	MAPK10/ SOCS1	Decreasing inflammation, lipid uptake and foam cell formation	(111–114)
miR-125a-5p	ORP9	Reduction of lipid uptake and secretion of inflammatory cytokines	(115)
miR-146a	TNF receptor (TNFR) associated factor 6 (TRAF6)	Decreasing intracellular LDL cholesterol content and inflammation	(116–118)
miR-33a/b	ABCA-1	HDL biogenesis and reverse cholesterol transport	(119, 120)
miR-34a	SirT1	Inducing endothelial cell senescence and inhibiting cell cycle	(121)
miR-10a	MAP3K7	Inhibits NF-kB activation and down regulation of inflammatory molecules	(122)
miR-302a	ABCA1	HDL biogenesis, cholesterol efflux and inhibiting foam cell formation	(77)
miR-126	VCAM-1	Inhibiting angiogenesis and inflammation	(123–125)
miR-21	MKK3	Cholesterol efflux and inhibiting foam cell formation	(21, 126)
miR-92a	STAT3	Increasing inflammation	(127)
miR-223	ICAM-1 and NLRP3	Reducing foam cell formation and production of proinflammatory cytokines	(128–130)

The overexpression of miR-146a can inhibit the activation of the TLR4 signaling pathway and prevent ox-LDL accumulation (116). Overexpression of miR-146a significantly decreases intracellular LDL cholesterol content and secretion of IL-6 and IL-8, chemokine (C-C motif) ligand-2, and MMP-9 in macrophages by targeting CD40L (116, 117). It was demonstrated that miR-146a regulates IL-1 receptor-associated kinase-1 (IRAK1) and TNF-receptor-associated factor-6 (TRAF6) (118). The protective role of miR-125a-5p against atherosclerosis was performed through regulating the pro-inflammatory response, lipid uptake by macrophages, decreasing content of the inflammatory cytokines: tumor growth factor-beta (TGF-α), TNF-α, IL-2, and IL-6 (115). The miR-125a-5p expression can cause the expression of LOX-1 and CD68 leading to a decrease of ox-LDL-stimulated macrophage uptake (115). Thus, it suppresses oxysterol binding protein like-9 (ORP9) and leads to reduction of lipid uptake and the secretion of inflammatory cytokines, including IL-2, IL-6, TNF-α, and TGF-β, in ox-LDL stimulated human primary monocytes (115). miR-223 is one of the most abundant miRs in LDL and HDL particles and inflammation (128). NOD-like receptor pyrin domain containing 3 (NLRP3) and ICAM-1 are the targets of miR-223. Upregulation of both these targets increases endothelial inflammation and causes leukocyte infiltration and inflammation connected with atherosclerosis (129, 130). It inhibits cholesterol biosynthesis via suppressing the sterol enzymes 3-hydroxy-3-methylglutaryl-CoA synthase 1 (HMGCS1) and methylsterol monoxygenase 1 (MSMO1) in humans (131). miR-10a up-regulation can influence inflammation through decreasing IκB/NF-κB activation, downregulation of inflammatory biomarkers, such as MCP-1, VCAM-1, E-selectin, IL-6, and IL-8. So, it suppresses inflammatory molecules in endothelial cells (122). miR-21 is a vital signaling mediator in an inflammatory state. Inhibition of this miRNA is related to the progression of atherosclerosis via increasing expression of mitogen-activated protein kinase kinase 3 (MKK3), inducing both p38 and the JNK (c-Jun N-terminal kinase) signaling pathway and regulating the expression of different pro-inflammatory mediators, including lipopolysaccharides and TNF-α (126, 132). miR-302a can regulate ABC transporters, which are involved in cholesterol efflux. The action mechanisms of this miRNA are increasing ABCA1 activity, cholesterol efflux out of macrophages, and preventing foam cell formation and growth of the atheromatous plaque (77). miR-126 can affect the inflammatory state of vasculature by the activation and infiltration of leukocytes via the vascular wall. It inhibits VCAM-1. Thus, miR-126 inhibition leads to enhancement of the proinflammatory TNF-α expression, the activity of NF-κB (nuclear factor κB), the activity of VCAM-1, as well as leukocyte-endothelial cells interactions and atherosclerotic lesions formation (123–125). Overexpression of miR-155 can cause attenuated inflammation and the subsequent foam cell formation via miR-155/calcium-regulated heat-stable protein 1 (CARHSP1)/TNF-α signaling pathway and targeting of mitogen-activated protein kinase 10 (MAPK10) signaling pathway (111, 112). It was suggested that miR-155 can contribute to the inflammatory processes through increasing STAT3 and nuclear factor kappa-light-chain-enhancer of activated B cells (NF-kB) signaling and targeting the suppressor of cytokine signaling 1 (SOCS1) in ox-LDL-induced macrophages (113). miR-155 could decrease the lipid uptake in ox-LDL-stimulated cells (114). miR-33a/b is a key regulator of lipid metabolism. It plays a key role in regulating reverse cholesterol transport by inhibiting the expression of ABCA1 at the RNA and protein level and decreasing cellular cholesterol efflux to apolipoprotein A-I (ApoA-I) (119, 120). Inhibition of miR-33a/b causes the following consequences: Increasing β-oxidation and decreasing fatty acid synthesis, ameliorating circulating lipids profile, and slowing down the progression of atherosclerosis (119, 120).

miR-92a expression in the endothelial cells of atherosclerosis-prone areas is an important regulator of atherosclerosis development via targeting STAT3 and secreting IL-6 and MCP-1 (127). miR-34a can develop atherosclerosis via inhibiting cell cycle and SirT1 protein expression, inducing endothelial cell senescence and repressing cell proliferation (121).

### Role of Medicinal Plants and Their Active Compounds in Atherosclerosis

**Figure 2:**
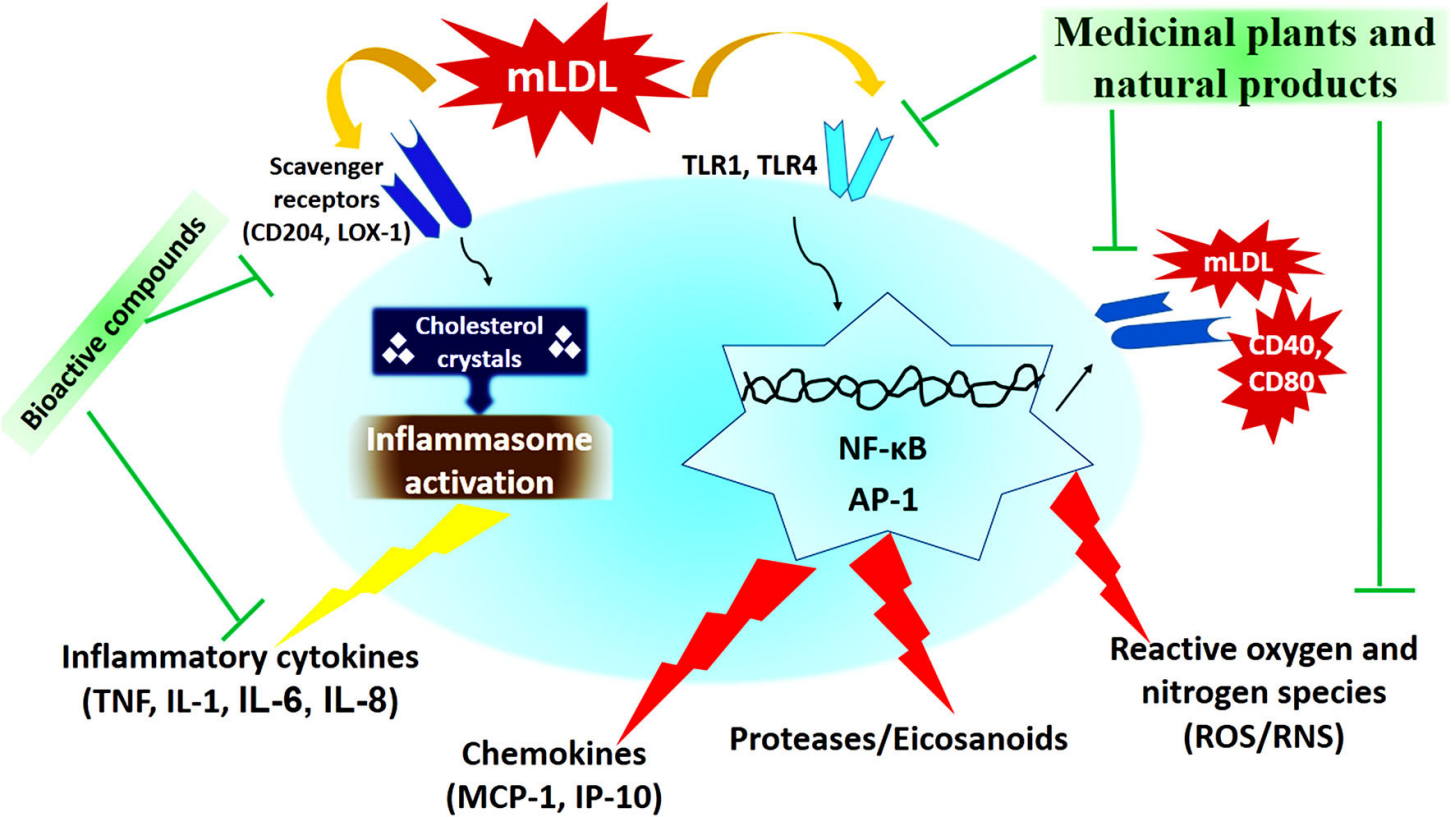
The cellular mechanism of atherosclerosis development and mechanisms of action of medicinal plants and natural products (173).

In recent decades, medicinal plants and natural products have been considered as one of the most important anti-atherosclerotic strategies for the treatment and prevention of atherosclerosis. Medicinal plants and natural products with their potential antioxidant, antiatherogenic, and anti-thrombotic properties, prevent atherosclerosis and its harmful complication through modulating the pathways of the inflammatory response, reducing cholesterolemia, free radicals, and decreasing vascular resistance (133, 134). The most important medicinal plants with anti-atherosclerotic actions have been summarized in Table 3 and Figure 2.

**Table 3:** Important anti-atherosclerotic medicinal plants and compounds.

**Medicinal plants**	**Active compounds**	**Action mechanism**	**Reference**
**Anti-inflammatory effects of medicinal herbs**			
*Salvia miltiorrhiza*	Salvianolic acid B and cryptotanshinone	Decreasing expression of MMP-9 and inhibition of NF-kB pathway and ICAM-1,VCAM-1 and MCP-1 expression	(135–137)
*Glycyrrhiza glabra*	glabridin	Blockage of JNK and NF-kB signaling, suppression of TNF-a, and production of IL-1b	(138–140)
*Allium sativum*	kaempferol	Inhibiting of inflammation signaling (like TNF-a, IL-1b, ICAM-1)	(141–145)
*Astragalus membranaceus*	Astragaloside IV	Down regulating of CD40L, CD40, CXCR4 and SDF-1	(146, 147)
*Punica granatum*	Ellagic acid and punicalagin	Decreasing plasma levels of IL-6 and TNF-α and increasing IL-10	(148–150)
*Magnolia offinicalis*	Magnolol	Suppressing phosphorylation of Tyr705, Ser727 and Tyr705 and stat3, inhibition of NF-kB pathway and inhibition of adhesion molecules expression	(151)
*Curcuma wenyujin*	β-Elemene	Inhibiting production of IL-1β, TNF-α, INF-γ, MCP-1, and ICAM-1	(152)
*Tripterygium wilfordii*	Celastrol	Decreasing production of iNOS, NO, and pro-inflammatory cytokines	(153)
*Ginkgo biloba*	*Ginkgo biloba* extract	Decreased expression of IL-1β,TNF-α and IL-10	(154)
*Curcuma longa*	Bisacurone	Inhibiting activity of iNOS, COX-2, lipoxygenase, and xanthine oxidase. Inhibiting JAK/STAT signaling pathway	(155)
*Coptis chinensis*	berberine	NF-kB activation and JNK phosphorylation	(156)
**Lipid-lowering effects**			
*Tribulus terrestris*	*Tribulus terrestris* extract	Decreasing total cholesterol, LDL-C, and triglyceride (TG)	(133, 157)
*Ocimum basilicum*	Aqueous extract of *Ocimum basilicum*	Decreasing total cholesterol, LDL-C, and triglyceride (TG)	(133, 158)
*Salvia miltiorrhiza*	Salvianolic acid B	Inhibiting CD36 and decreasing total cholesterol, LDL-C, and triglyceride (TG)	(159)
*Celastrus orbiculatus*	*Celastrus orbiculatus* extract	Up regulating SR-B1, CYP7A1 and HMG-CoA, decreasing total cholesterol, LDL-C, and triglyceride (TG)	(160)
*Panax notoginseng*	saponins	Decreasing total cholesterol, LDL-C, and triglyceride (TG)	(161)
*Nigella sativa*	Propolis and thymoquinone	Decreasing total cholesterol, LDL-C, and triglyceride (TG)	(162, 163)
*Astragalus membranaceus*	Astragaloside IV	Increased activity of PPARα and PPARγ	(146, 147)
*Allium sativum*	Flavonoids, alkaloids	Decreasing total cholesterol, LDL-C, and triglyceride (TG)	(141–145)
**Anti-oxidative effects and inhibitory activity of LDL oxidation**			
*Nigella sativa*	Propolis and thymoquinone	Scavenging of free radicals	(162, 163)
*Salvia miltiorrhiza*	Danshenol A, Tanshinone IIA, Salvianolic acid B and Cryptotanshinone	Inhibiting ROS production and decreasing LOX-1 expression	(135, 164)
*Punica granatum*	Phenol, Ellagic acid and punicalagin	Decreasing ROS production	(148–150)
*Buddleja officinalis*	Aqueous extract of *B. officinalis*	Inhibiting ROS production	(156)
*Curcuma wenyujin*	β-Elemene	Inhibiting ROS production and eNOS expression	(152)
*Celastrus orbiculatus*	*C. orbiculatus* extract	Inhibiting ROS production	(160)
*Tripterygium wilfordii*	Celastrol	Inhibiting ROS production and LOX-1 and iNOS expression	(153)
*Ocimum basilicum*	Aqueous extract of *Ocimum basilicum*	Inhibiting Radical anion superoxide production	(133, 158)
*G. glabra*	Glycyrrhizin and glabridin	Inhibiting ROS production, inhibiting LDL NADPH oxidase and preventing cholesterol oxidation	(138–140)
*A. sativum*	Flavonoid, kaempferol	Decreasing lipid peroxidation, superoxide and hydroxyl radicals	(141–145)
*Sesamum indicum*	Sesamolinol, sesamol	Inhibiting ADP-Fe+/NADH-induced peroxidation and lipid oxidation	(165, 166)
**Suppression of cholesterol accumulation and foam cell formation**			
*Curcuma longa*	curcumin	Promoting ABCA1-dependent cholesterol efflux and inhibiting of SR-A-mediated oxidized LDL uptake	(167–169)
*Coptis chinensis*	berberine	Increasing LXRa-ABCA1-dependent cholesterol efflux, activating the AMPK-SIRT1-PPAR-?pathway and down-regulating HMGR expression	(156)
*Allium sativum*	kaempferol	Down-regulation of HMGR, FAS, SREBP-1c, G6PDH and acetyl-CoA carboxylase	(170)
*Salvia miltiorrhiza*	Salvianolic acid B	Down-regulating of CD36 expression	(159)
*Punica granatum*	Ellagic acid and punicalagin	Up-regulation of ABCA1 expression and regulating PPAR-ABCA1 pathway	(148–150)
**Inhibitory effects of medicinal herbs against monocyte recruitment**			
*Purple perilla*	Purple perila extract and α-asarone	Inhibiting foam cell formation and SR-B1 expression, upregulation of ABCA1 and ABCG1	(133, 171)
*Buddleja officinalis*	Aqueous extract of *Buddleja officinalis*	Inhibiting VCAM-1 and ICAM-1 expression	(156)
*Curcuma longa*	curcumin	Inhibiting IκBα, Akt, and PKC phosphorylation and VCAM-1 expression	(167–169)
*Salvia miltiorrhiza*	Cryptotanshinone	Reducing LOX-1, VCAM-1 and ICAM-1 expression	(136, 164)
*Paeonia lactiflora*	Paeonol	Inhibiting ICAM- 1 expression and NF-κB p65 translocation into the nucleus	(172)
*Panax notoginseng*	Saponins	Inhibiting ICAM- 1 and VCAM-1 expression	(161)

The experimental New Zealand rabbits group treated with extract of *Tribulus terrestris* revealed decreased levels of total cholesterol (TC), high-density lipoprotein cholesterol (HDL-C), low-density lipoprotein cholesterol (LDL-C), and triglyceride (TG) in serum compared to the control group (133, 157). In Triton WR-1339-induced hyperlipidemic rats treated with *Ocimum basilicum* extract, the TC, TG, and LDL-C levels decreased (133, 158). *Salvia miltiorrhiza* with its Salvianolic acid B can inhibit CD36-mediated lipid uptake via binding directly to CD36 with high affinity (159). *Salvia miltiorrhiza* and cryptotanshinone decrease the expression of MMP-9, NF-κB, and the production of adhesion molecules. This plant prevents the migration of human aortic smooth muscle cells (174, 175). *Salvia miltiorrhiza* and its active compound, Danshenol, suppress ICAM-1 expression and relevant monocyte adhesion to endothelial cells through the NADPH oxidase subunit 4 (NOX4)-dependent inhibitor of kappa B (IκB) kinase β (IKKβ)/nuclear factor-kappa B (NF-κB) pathway (164). Cryptotanshinone from *S. miltiorrhiza* decreased LOX-1, ICAM-1, and VCAM-1 expression (135, 136). Salvianolic acid B from *Salvia miltiorrhiza* reduced JAK2 (Tyr 1007/1008) and STAT1 (Tyr701 and Ser727) phosphorylation. It enhanced protein inhibitor of activated STAT 1 (PIAS1) and suppressor of cytokine signaling 1 (SOCS1) expressions in endothelial cells (176). The modulatory effects of salvianolic acid B and Cryptotanshinone, bioactive compounds from *S. miltiorrhiza*, significantly suppressed the atherosclerotic plaque formation by inhibiting the LOX-1 and MMP-9 expression and affecting PI3K/Akt, MAPK, NF-KB pathways (135, 137). *Allium sativum* with large amounts of flavonoids, such as kaempferol inhibits cyclooxygenase and lipoxygenase and prevents the accumulation of thrombocytes. *Allium sativum* extract decreases the level of malondialdehyde, superoxide, and hydroxyl radicals (141). *Allium sativum* has antithrombotic activity by suppressing cyclooxygenase, reducing the generation of thromboxane B2, prostaglandin E2, and leukotriene C4 by platelets (142). Extract from *Allium sativum* inhibits platelet aggregation via different mechanisms including increasing cyclic nucleotides, inhibiting GPIIb/IIIa receptor and fibrinogen binding, and preventing phosphorylation of collagen-induced ERK, JNK, and p38 (143–145). It lowers blood lipids such as total cholesterol and triglycerides through down-regulating acetyl-CoA carboxylase (ACC), acyl-CoA cholesterol acyltransferase (ACAT), HMGR, fatty acid synthase (FAS), sterol regulatory element-binding protein-1c (SREBP-1c), and glucose-6- phosphate dehydrogenase (G6PD) (170). *Nigella sativa* and its main active compounds, propolis and thymoquinone, prevent LDL oxidation and decrease in the development of atherosclerotic lesions. It scavenges the free radicals and has antioxidant activity. This medicinal plant and its active compounds decreased TC, LDL-C, and TG, while increased HDL-C levels in hypercholesterolemic rabbits (162, 163). *Celastrus orbiculatus* up-regulated scavenger receptor class B type 1 (SR-B1), cholesterol 7α-hydroxylase A1 (CYP7A1), and 3-hydroxy-3-methyl-glutaryl-coenzyme A (HMG-CoA) reductase and significantly decreased TC, non-HDL-C, TG, and lipid deposition in the arterial wall (160). *C. orbiculatus* decreased CRP, IL-6, and TNF-α levels in plasma and CD68 up-regulation and NF-κB p65 protein activation in the arterial wall (160). *Magnolia officinalis*, with its main compound, magnolol, suppressed IL-6-induced phosphorylation of Tyr705 and Ser727 on STAT3. STAT3 is a transcription factor involved in inflammatory responses. It reduces ICAM-1 expression on the endothelial surface (151). The ethanolic extract of *Astragalus membranaceus* decreased blood glucose and triglyceride (TG) via the increasing activity of PPAR-α and PPAR-γ (146). Also, downregulation of CD40 ligand and C-X-C chemokine receptor type 4 (CXCR4) expression on the platelet surface, and stromal cell-derived factor-1 (SDF-1) and CXCR4 expression in the aorta are the most important effects of *A. membranaceus* and Astragaloside IV (147). β-Elemene isolated from *Curcuma wenyujin* inhibits the production of pro-inflammatory cytokines and cell adhesion molecules such as IL-1β, TNF-α, INF-γ, MCP-1, and ICAM-1 and decreases the size of atherosclerotic lesions (152). *Tripterygium wilfordii* and its triterpenoid, Celastrol, prevent phosphorylation and degradation of IκB. This medicinal plant reduces the production of inducible nitric oxide synthase (iNOS), NO, and pro-inflammatory cytokines such as TNF-α and IL-6 (153). *Curcuma longa* and curcumin inhibit SR-A-mediated oxidized LDL uptake and lead to reduced cholesterol accumulation in the arterial wall via activation of the AMPK-SIRT1- LXRa signaling pathway (167, 168). Also, curcumin shows anti-inflammatory properties via inhibiting the activity of iNOS, COX-2, lipoxygenase, and xanthine oxidase, and activating NF-kB (169). *C. longa* inhibits IκBα, protein kinase B (Akt), and protein kinase C (PKC) phosphorylation and suppresses VCAM-1 expression (155). *Glycyrrhiza glabra* and its main flavonoid, glabridin, have anti-inflammatory properties via different action mechanisms including inhibiting TNF-a-stimulated gene expression of VCAM-1 and ICAM-1 and blocking JNK and NF-kB (138, 139). It prevents LDL oxidation through inhibiting 2, 2-azobis (2-amidinopropane) hydrochloride (AAPH)–stimulated production of cholesteryl linoleate hydroperoxide in LDL (140). *Coptis chinensis* and its main compound, berberine, increase LXRa-ABCA1-dependent cholesterol efflux and activate the AMPK-SIRT1-PPAR-g pathway, consequently decreasing foam cell formation (156). *Punica granatum*, ellagic acid, and punicalagin, possess obvious anti-inflammatory effects including reducing plasma levels of IL-6 and TNF-α, increasing the anti-inflammatory cytokine IL-10, and decreasing the translocation of NF-kB from the cytosol to the nucleus (148–150). *Ginkgo biloba* extract decreases IL-1β, TNF-α, IL-10 expression, and growth of vascular smooth muscle cells (VSMC) (154).

Paeonol, the active compound of *Paeonia lactiflora*, decreased ICAM- 1 expression via phosphorylation of IκBα and inhibition of NF-κB p65 translocation into the nucleus. It had influences on extracellular signal-regulated kinase (ERK) induced by TNF-α and blocked the phosphorylation of p38 (172). Saponins, the active compounds of *Panax notoginseng* suppress TNF-α-induced endothelial adhesion molecules such as ICAM-1 and VCAM-1 expression and reduce monocyte adhesion to the endothelium (161). *Purple perilla* and its main compound α-asarone prevent ox-LDL-induced foam cell formation via inhibiting SR-B1 expression. Also, *Purple perilla* causes the adenosine triphosphate (ATP)-binding cassette transporter A1 (ABCA1) and ABCG1 upregulation, and subsequently result in cholesterol efflux from macrophages through interactions between peroxisome proliferator-activated receptorγ (PPARγ), liver X receptor α (LXRα), and ABC transporters (133). *Buddleja officinalis* reduces VCAM-1 and ICAM-1 through inhibition of NF-κB and reactive oxygen species (ROS) (156). *Sesamum indicum* with its antioxidant and anti-inflammatory properties showed inhibitory effects on membrane microsomal peroxidation, lipid peroxidation, ADP-Fe^3+^/NADH-induced peroxidation, and Cu ions-induced LDL oxidation. Hence, this plant decreases the levels of plasma triglyceride and cholesterol, and LDL-cholesterol (LDL-C) (165, 166).

## Conclusions

Atherosclerosis is a chronic inflammatory disease that is continuous crosstalk between the lipid metabolism and immune-inflammatory pathways. Accumulation of intracellular lipids is a fundamental event in atherogenesis at the cellular level. The accumulation of intracellular modified lipids, especially ox-LDL, leads to foam cell formation. Inflammation participates in all phases of atherosclerosis. Lipid droplets accumulate in the cytoplasm of Pericytes, macrophages, and smooth muscle cells and lead to change their appearance. All risk factors of atherosclerosis cause an inflammatory response. Inflammatory cytokines cause the accumulation of intracellular lipids. Intracellular lipids accumulation leads to rupture of the cells. As a result of the increase of pro-inflammatory cytokines, endoplasmic reticulum stress in the arterial wall cells and apoptosis occur. This process and cytokine-induced inflammation lead to interrupt the normal activity of mitochondria and then impaired mitophagy and apoptosis. Thus, both lipoprotein metabolism and inflammatory immune response play vital roles in the initiation, perpetuation, and eventually, resolution of the atherosclerosis process.

This information leads to the development of new approaches to the diagnosis and treatment of atherosclerosis such as evaluation of microRNAs and lncRNAs. On the other hand, new developments based on using cell models, medicinal plants, and natural products as therapeutic tools have been developed to prevent lipid accumulation and foam cell formation. Recently, lncRNAs and miRNAs have been considered for the treatment and prevention of atherosclerosis. Hence, the therapeutic effects of these molecules, especially lncRNAs, have largely remained experimental at this time. Expressing the therapeutic effect of these molecules with more details and their mechanism of action needs more experiments and research in the future. Totally, various lncRNAs and miRNAs can prevent and treat atherosclerosis by regulating HDL biogenesis, cholesterol efflux, lipid metabolism, regulating smooth muscle proliferation, and controlling inflammation.

Medicinal plants and their active compounds can prevent atherosclerosis through the following mechanisms: in fact, medicinal plants decrease total cholesterol, LDL-C, and TG. Various bioactive compounds reduce LOX-1 expression. They inhibit ROS production. Natural products inhibit the foam cell formation and SR-B1 expression. Also, the production of IL-1β, TNF-α, INF-γ, MCP-1, and ICAM-1 is inhibited by different medicinal plants.

## Author Contributions

KM and MR-K: conceptualization. MR-K: methodology and supervision. KM, EB, and MR-K: writing—original draft preparation and writing—review and editing. All authors contributed to the article and approved the submitted version.

## Conflict of Interest

The authors declare that the research was conducted in the absence of any commercial or financial relationships that could be construed as a potential conflict of interest.

## Publisher's Note

All claims expressed in this article are solely those of the authors and do not necessarily represent those of their affiliated organizations, or those of the publisher, the editors and the reviewers. Any product that may be evaluated in this article, or claim that may be made by its manufacturer, is not guaranteed or endorsed by the publisher.

## Abbreviations

ABCA1: ATP binding cassette transporter A1
ABCG1: ATP-binding cassette transporters G1
ApoB: apolipoprotein B
CIC: circulating immune complexes
CRP: C-Reactive Protein
EC: endothelial cell
EndMT: endothelial-to-mesenchymal transition
eNOS: endothelial nitric oxide synthase
HDL-C: high-density lipoprotein cholesterol
ICAM: Intercellular Adhesion Molecule-1
IDL: intermediate-density lipoproteins
IFN-γ: γ-interferon
IL-8: Interleukin-8
JNK: cJun NH2-terminal kinase
LDL: low-density lipoprotein
LDL-C: low-density lipoprotein cholesterol
lncRNA: long non-coding RNA
LOX-1: the lectin-like oxidized LDL 1 receptor
Lp-PLA2: lipoprotein-associated phospholipase A2
LPL: lipoprotein lipase
LPS: lipopolysaccharide
LRP1: low-density lipoprotein receptor-related protein 1
lysoPC: lysophosphatidylcholine
M-CSF: Macrophage Colony-Stimulating Factor
MALAT1: metastasis-associated lung adenocarcinoma transcript 1
MCP-1: Monocyte Chemotactic Protein-1
mLDL: modified low-density lipoprotein
MMP: Matrix Metalloproteinase
NEAT1: nuclear enriched abundant transcript
NF-kB: nuclear transcription factor-kB
NFIA: Nuclear factor I A
ox-LDL: oxidized low-density lipoprotein
PAR: Protease Activated Receptor
RNS: reactive nitrogen species
ROS: Reactive oxygen species
S-SMase: sphingomyelinase
SMCs: Smooth Muscle Cells
SR: scavenger receptors
SR-BI: scavenger receptor class B type I
SR-E1: the lectin-like oxidized LDL 1 receptor
SRC: non-receptor tyrosine kinase
STAT3: signal transducer and activator of transcription 3
TC: total cholesterol
TG: triglyceride
TLR4: Toll-like receptor 4
TNF-β: tumor necrosis factor β
VCAM: Vascular Cell Adhesion Molecule-1
VLDL: very-low-density lipoproteins
VSMCs: vascular smooth muscle cells.
